# Functional characterization of biodegradable nanoparticles as antigen delivery system

**DOI:** 10.1186/s13046-015-0231-9

**Published:** 2015-10-06

**Authors:** A. Petrizzo, C. Conte, M. Tagliamonte, M. Napolitano, K. Bifulco, V. Carriero, A. De Stradis, M. L. Tornesello, F. M. Buonaguro, F. Quaglia, L. Buonaguro

**Affiliations:** Laboratoy Molecular Biology and Viral Oncology, Department of Experimental Oncology, Istituto Nazionale per lo Studio e la Cura dei Tumori “Fondazione Pascale” - IRCCS, Via Mariano Semmola 142, 80131 Naples, Italy; Department of Pharmacy, University of Napoli Federico II, Via Domenico Montesano 49, 80131 Naples, Italy; Laboratory of Clinical Immunology, Istituto Nazionale per lo Studio e la Cura dei Tumori “Fondazione Pascale” - IRCCS, Via Mariano Semmola 142, 80131 Naples, Italy; Laboratory of Tumor Progression, Istituto Nazionale per lo Studio e la Cura dei Tumori “Fondazione Pascale” - IRCCS, Via Mariano Semmola 142, 80131 Naples, Italy; National Research Council Institutional Sustainable Plant Protection, Bari, Italy

**Keywords:** PLGA nanoparticles, PLGA/PEI nanoparticles, Antigen delivery system, Cancer vaccine

## Abstract

**Background:**

Peptide based vaccines may suffer from limited stability and inefficient delivery to professional antigen-presenting cells (APCs), such as dendritic cells (DCs). In order to overcome such limitations, several types of biodegradable nanoparticles (NPs) have been developed as carrier system for antigens. The present study describes for the first time the extensive biological characterization of cationic NPs made of poly (D,L-lactide-co-glycolide) (PLGA) and polyethylenimine (PLGA/PEI) as delivery system for protein/peptide antigens, with potential in therapeutic cancer vaccine development.

**Results:**

Flow cytometry as well as confocal laser scanning microscopy (CLSM) showed that PLGA/PEI NPs are more readily taken up than PLGA NPs by both human CD14^+^ monocytes and mouse Hepa 1–6 hepatoma cell line. No signs of toxicity were observed in either cellular setting. Sequential image acquisition by TEM showed an intracellular apical localization for PLGA NPs and a perinuclear localization for PLGA/PEI NPs. Both NPs showed a clathrin-dependent as well as a caveolin-dependent internalization pathway and, once in the cells, they formed multivesicular endosomes (MVE). Finally, an *ex vivo* priming experiment showed that PLGA/PEI NPs are comparable to PLGA NPs in delivering a non-self antigen (i.e., ovalbumin - OVA) to immature dendritic cells (imDCs), which matured and induced autologous naïve CD4^+^ T cells to differentiate to memory (i.e., central memory and effector memory) cells. Such a differentiation was associated with a Th1 phenotype suggesting a downstream activation and amplification of a CD8^+^ T cell cytotoxic response. The same OVA antigen in a soluble form was unable to induce maturation of DCs, indicating that both NP formulations provided an intrinsic adjuvanting effect combined to efficient antigen delivery.

**Conclusions:**

Our study represents the first report on side-by-side comparison of PLGA and PLGA/PEI NPs as strategy for protein antigen delivery. PLGA/PEI NPs are superior for cellular uptake and antigen delivery as compared to PLGA NPs. Such an evidence suggests their great potential value for vaccine development, including therapeutic cancer vaccines.

**Electronic supplementary material:**

The online version of this article (doi:10.1186/s13046-015-0231-9) contains supplementary material, which is available to authorized users.

## Background

Our improved understanding of antigen recognition and presentation by professional APCs to effector lymphocytes has led to the development of several cancer vaccine concepts, most of which based on peptides [1].

Cancer vaccines based on autologous DCs loaded *ex vivo* with peptides have been developed and tested in several clinical trials with controversial results (reviewed in [2, 3]) due to several reasons, including the variable efficiency of the generated DCs in antigen presentation [4].

Recently, biocompatible PLGA NPs have generated tremendous interest in antigen delivery due to several features, including 1) antigen protection from early degradation in biological environments [5]; 2) prolonged antigen presentation by DCs for induction of B and T cell response after both systemic and mucosal administration [6]; 3) antigen reservoir for stimulation of CTL response [7]. However plain PLGA NPs have suboptimal adjuvant properties *in vivo* resulting in poor DC maturation [8].

Several strategies have been developed so far to enhance NP uptake by DCs, including surface decoration with ligands of DC receptors as well as modification of surface charge with polycations able to promote electrostatic interactions with anionic cell membranes [9]. The latter approach was found to be successful when PLGA NPs were modified with protamine that enhanced the NP properties of inducing activation and maturation of bone-marrow DCs [10, 11].

In this perspective, polycations may represent a promising option in “nanovaccine” approaches to modify surface properties of PLGA NPs. Indeed, polyethylenimine (PEI) has been recently shown to have anticancer/immunostimulatory activities [12] as well as adjuvant properties for glycoprotein antigens administered at mucosal sites [13].

On these premises, in the present study we developed NPs made of PLGA, alone or in association with PEI, to assess whether the modification would result in enhancement of both NP cellular uptake and presentation of the carried non-self OVA antigen [14].

The rate of uptake and intracellular distribution of both NPs was assessed in a murine Hepa 1–6 hepatoma cell line and in human PBMCs.

Finally, an *ex vivo* priming experiment showed that both NPs were efficient in delivering the OVA antigen to immature dendritic cells (imDCs), inducing their maturation. Such matured DCs (mDCs) were able to prime autologous naïve CD4^+^ T cells to differentiate into memory T cells with a Th1 phenotype. This is suggestive of a downstream activation and amplification of a CD8^+^ T cell cytotoxic response, which is the relevant one in a therapeutic cancer vaccine setting.

## Results

### Nanoparticle properties

Properties of the produced NPs are reported in Table 1. The size of NPs was increased by OVA entrapment only in the case of PLGA NPs. Negative zeta potential of PLGA NPs switched to positive values in the case of PLGA/PEI NPs, suggesting that PEI was partly present onto NP surface. Lower entrapment efficiency of OVA was found for OVA-PLGA/PEI NPs, presumably due to the occurrence of electrostatic interactions between OVA and PEI, resulting in both localization of an OVA fraction onto NP surface and escape from the polymer matrix to the external phase during particle formation.

**Table 1:** Properties of unloaded and OVA-loaded NPs. SD were calculated on three different batches

Code	OVA % w/w	Mean DH nm ± SD	P.I.	Zeta potential mV ± SD	PEI loading mg/100 mg NPs	OVA actual loading mg/100 mg NPs (Entrapment efficiency %)^a^
PLGA NPs	-	175 ± 12	0.165	−32.8 ± 5.6	-	-
OVA-PLGA NPs	4	253 ± 16	0.166	−32.1 ± 2.0	-	3.6 ± 0.7 (92.9 ± 17.4)
PLGA/PEI NPs	-	172 ± 5	0.162	+36.3 ± 4.0	6.2	-
OVA-PLGA/PEI NPs	4	161 ± 9	0.147	+42.9 ± 5.3	6.6	3.3 ± 0.4 (77.5 ± 10.3)

^a^Ratio between actual and theoretical loading x 100

**Fig. 1:**
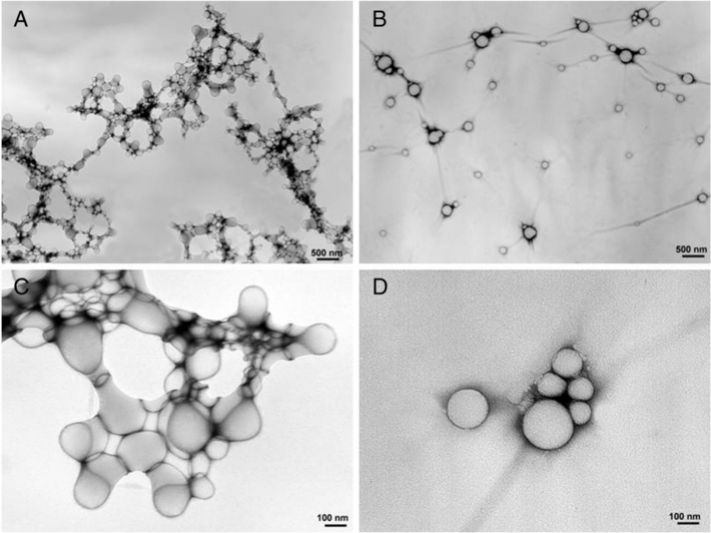
TEM morphology study. TEM analysis of PLGA (**a** and **c**) and PLGA/PEI (**b** and **d**) NPs

TEM analysis of NP morphology showed monolayer spheroid particles with a diameter ranging from 100 to 200 nm for PLGA NPs (Fig. 1a and c) and 50 to 250 nm for PLGA/PEI NPs (Fig. 1b and d) in line with values observed by PCS.

**Fig. 2:**
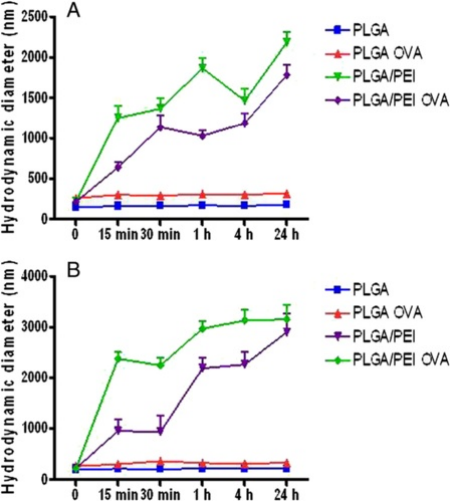
NP stability in cell culture media. Stability of NPs was analyzed at 37 °C in DMEM (**a**) and RPMI 1640 (**b**), respectively, by monitoring size by PCS. Data are reported as mean of three independent experiments ± SD

NP stability in both DMEM and RPMI culture media was analyzed. Both unloaded and OVA-PLGA NPs showed a satisfactory stability in the media. Conversely, the presence of PEI induced a remarkable aggregation of the NPs, likely due to interactions with serum proteins (Fig. 2a and b).

### NP uptake in human PBMCs and mouse Hepa 1–6 cell line

Cellular uptake studies were performed in a murine Hepa 1–6 hepatoma cell line and in human PBMCs. Fluorescently-labeled NPs prepared from a Rhodamine-PLGA conjugate (Additional file 1: Table S1) were employed for cellular uptake studies.

**Fig. 3:**
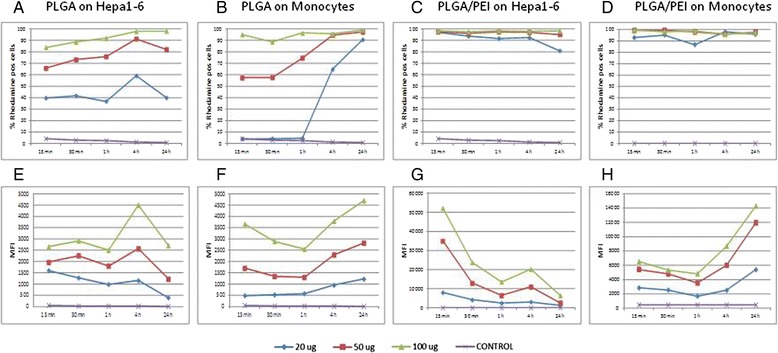
NP uptake in human PBMCs and mouse Hepa 1–6 cell line. Percent of Hepa 1–6 (**a** and **c**) and monocytes (**b** and **d**) which uptake PLGA-Rhod NPs (**a** and **b**) and PLGA-Rhod/PEI NPs (**c** and **d**) at different time points. Mean fluorescence intensity (MFI) of Hepa 1–6 (**e** and **g**) and monocytes (**f** and **h**) loaded with different concentration of PLGA-Rhod NPs (**e** and **f**) and PLGA-Rhod/PEI NPs (**g** and **h**), at different time points

The percentage of cellular uptake was evaluated at sequential time points, loading both cell types with increasing doses of both NPs (20, 50 and 100 μg/mL) (Fig. 3). Uptake of PLGA-Rhod NPs showed a dose- and time-dependent curve, reaching the saturation with the highest dose (i.e., 100 μg/mL) in Hepa 1–6 cells, and with all three doses in PBMCs, after 4–24 h (Fig. 3a and b). On the contrary, cellular uptake of PLGA-Rhod/PEI NPs in both cell types showed a dose- and time-independent curve, reaching the saturation as soon as 15 min, regardless the amount of NPs used for induction (Fig. 3c and d).

Moreover, the pattern of intracellular fluorescence showed a dose response curve in both cell types with both NPs (Fig. 3e-h). Considering the Hepa 1–6, for each of the three doses, the cellular fluorescence induced by PLGA-Rhod NP uptake was characterized by an initial lag phase, with a spike at 4 h, followed by a steep decline at 24 h (Fig. 3e), while the fluorescence induced by PLGA-Rhod/PEI NP uptake peaked as soon as at 15 min, with a MFI (mean fluorescence intensity) >20 fold than with PLGA-Rhod NPs, to sharply reduce at subsequent time points, with a small rebound at 4 h. This finding was consistently observed for all three doses (Fig. 3g).

CD14^+^ monocytes showed a different profile (Fig. 3f and h). Indeed, the cellular fluorescence induced by uptake of PLGA-Rhod NPs progressively increased at different time points with the lowest dose (20 μg/mL), while it progressively decreased for the first hour and sharply increased at 4 and 24 h with the higher doses (Fig. 3f). Moreover, unlike Hepa 1–6 cell line, the cellular fluorescence induced by uptake of PLGA-Rhod/PEI NPs in CD14^+^ monocytes was characterized by a “parabola-like” pattern similar to the one observed for PLGA-Rhod NPs, with a sharp increase at 24 h reaching >3 fold MFI values (Fig. 3h).

### Analysis of intracellular NP distribution by CLSM

Intracellular nanoparticle distribution was evaluated at different time points by fluorescence microscopy and CLSM in Hepa 1–6 cells and PBMCs loaded with 50 μg/mL of PLGA and PLGA-Rhod/PEI NPs.

**Fig. 4:**
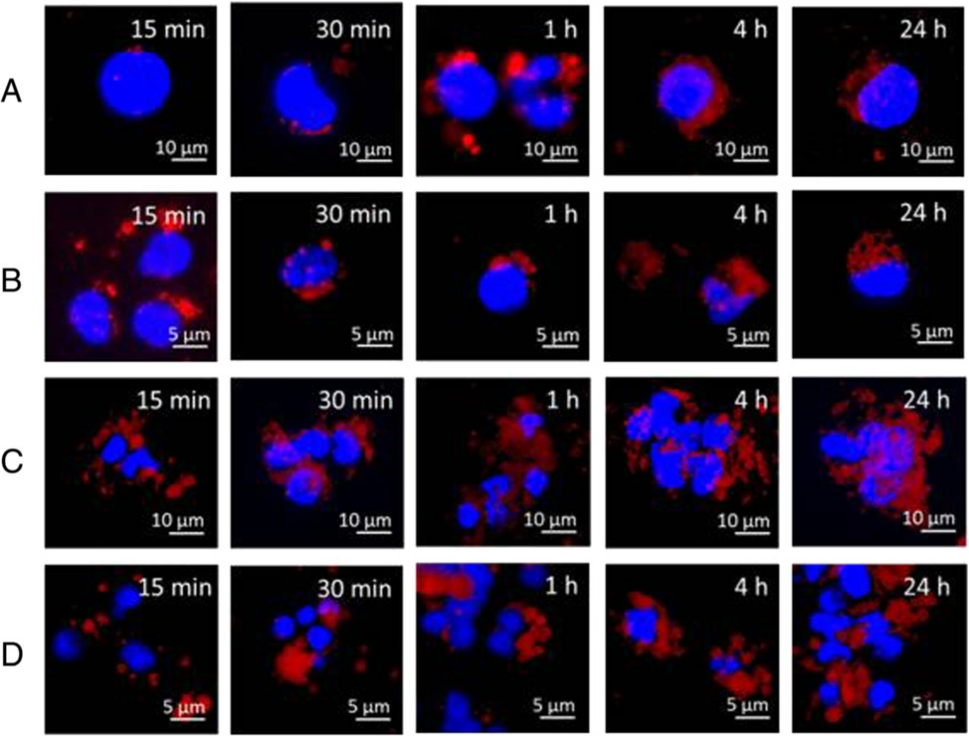
Intracellular NP distribution evaluated by fluorescence microscopy. Hepa 1–6 (**a** and **c**) and monocytes (**b** and **d**) were loaded with either 50 μg/mL PLGA-Rhod NPs (**a** and **b**) or PLGA-Rhod/PEI NPs (**c** and **d**) at different time points. Original magnification: 400X

The fluorescence microscopy analysis showed that PLGA-Rhod NPs exhibited a low aggregation profile and a time-dependent internalization in both cell types as indicated by the appearance, already after 30 min, of punctuate red fluorescent spots that progressively pervaded the entire cytoplasm (Fig. 4a and b). Conversely, an increase in size of NPs due to their aggregation was observed in both cell types loaded with PLGA-Rhod/PEI NPs. A massive internalization of clustered PLGA-Rhod/PEI NPs was observed already after 1 h incubation, in both Hepa 1–6 and PBMCs (Fig. 4c and d). Such observations are fully in agreement with data on hydrodynamic diameter (Fig. 2a and b).

**Fig. 5:**
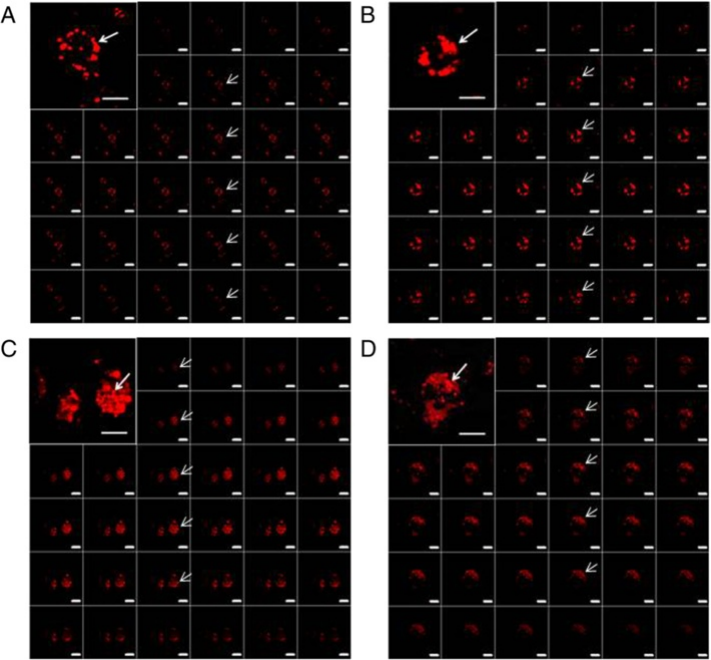
Analysis of intracellular NP distribution by CLSM. monocytes were loaded with 50 μg/mL PLGA NPs at different time points (**a** 30 min; **b** 1 h; **c** 4 h; **d** 24 h). Z-series images represent focal planes corresponding to 0.1 μm vertical interval. Internalization areas are indicated by arrows. Original magnification: 630X. Scale bar: 10 μm and 5 μm

The time dependent internalization of PLGA-Rhod NPs by monocytes exposed to 50 μg/mL PLGA-Rhod NPs for 30 min, 1 h, 4 h and 24 h was further confirmed by z-stack analysis of confocal images taken through the cell at 0.1 μm intervals (Fig. 5a-d). Internalization of PLGA-Rhod NPs occurred already after 30 min (Fig. 5 a) and increased after 1 h (Fig. 5b) and 4 h (Fig. 5c). After 24 h, cells showed a diffuse intra-cytoplasmic fluorescence together with smaller fluorescent spots, suggesting that, at this time point, escape from endolysosomes of PLGA-Rhod NPs might occur (Fig. 5d). The analysis of cells loaded with PLGA-Rhod/PEI NPs was not possible because of extensive cellular aggregation.

### Analysis of NP intracellular compartmentalization by TEM

**Fig. 6:**
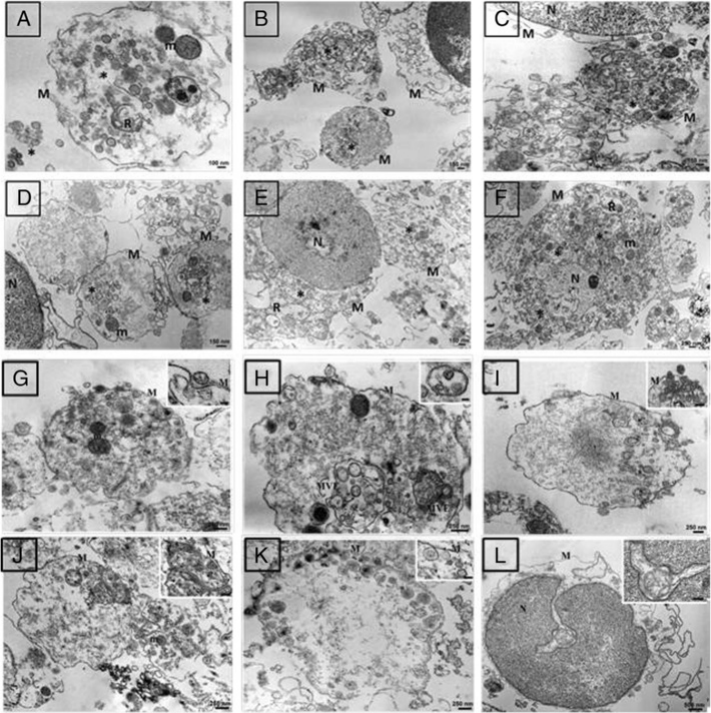
TEM ultrastructural study. Monocytes were loaded at different time points with either 50 μg/mL PLGA NPs (**a** 15 min; **b** 30 min; **c** 24 h) or PLGA/PEI NPs (**d** 15 min; **e** 30 min; **f** 24 h). Clathrin-dependent (**g** and **i**) and caveolin-dependent (**j** and **k**) pathways; multivesicular enodosomes (MVE) (**h**); interactions between vesicles and nuclear membrane (**l**) at 24 h post loading. NP internalization is indicated by asterisks. M: cytoplasmic membrane, N: nucleus, R: endoplasmic reticulum, m: mitochondria

TEM analysis of PBMCs showed a significantly different pattern of intracellular localization according to the NP formulation loaded. PLGA NPs were internalized as single particles and localized mostly at the cellular apical area for the entire incubation period (Fig. 6a-c). On the contrary, PLGA/PEI NPs were internalized as multiple particles, confirming particle-to-particle interactions found in the presence of proteins and more extensive uptake observed by flow cytometry as well as CLSM. Moreover, PLGA/PEI NPs were massively internalized already at 15 min post treatment (Fig. 6d), moved towards cellular core areas at 30 min (Fig. 6e) and reached the perinuclear region at 24 h (Fig. 6f). Both NPs showed a clathrin-dependent (Fig. 6g and i) and a caveolin-dependent (Fig. 6j and k) internalization pathway [15] and, once in the cells, they formed multivesicular endosomes (MVE) (Fig. 6h) [16]. Moreover, PBMCs loaded with PLGA PEI NPs often showed interactions between vesicles and nuclear membrane (Fig. 6l).

### Effect of NPs on *ex vivo* activation of naïve CD4^+^ T cells

MDDCs obtained from human PBMCs by standard procedure [17] were pulsed with 1.55 and 7.75 μg OVA (1X and 5X dose), either soluble or encapsulated in NPs. Loaded cells did not show any significant toxicity due to NP loading, as assessed by MTT assay (data not shown).

Half of the immature DCs pulsed with the two doses of OVA (OVA-imDCs) were further matured by a 48 h treatment with LPS (200 ng/mL), resulting in mature DCs (OVA-mDCs). Both OVA-imDCs and OVA-mDCs were used to prime autologous naïve CD4^+^ T cells labeled with CFSE in a 1:5 ratio.

**Fig. 7:**
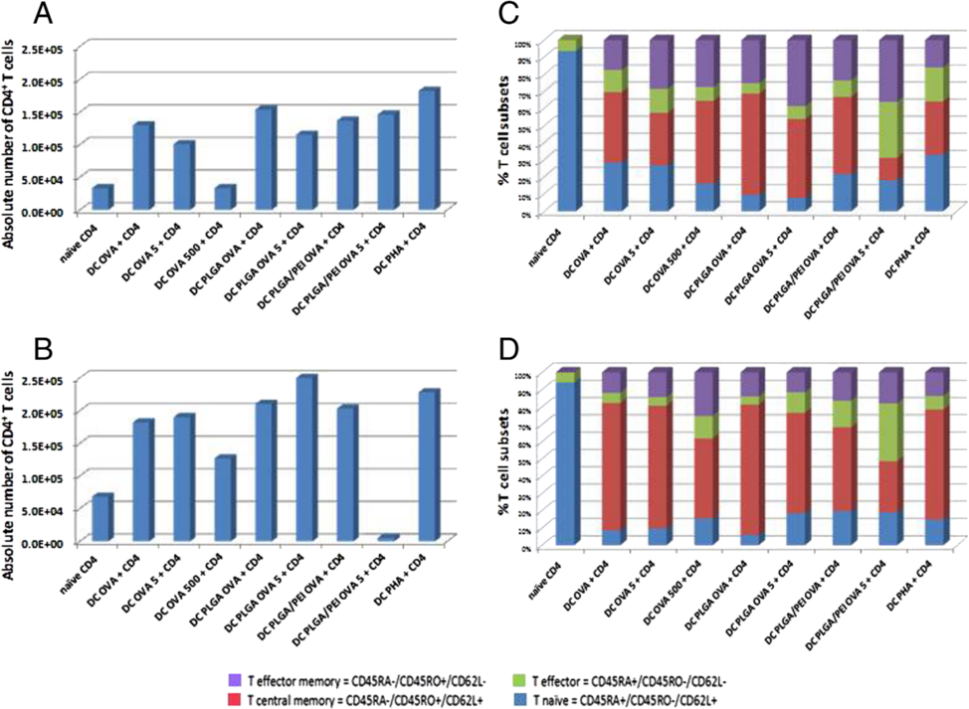
Effect of NPs on *ex vivo* differentiation of naïve CD4^+^ T cells. Absolute number of CD4^+^ T cells after 7 days of co-cultivation with either OVA-imDCs (**a**) or OVA-mDCs (**b**). CD4^+^ T cell differentiation after 7 days of co-cultivation with either OVA-imDCs (**c**) or OVA-mDCs (**d**). Naïve unstimulated CD4^+^ T cells represent basal activation. CD4^+^ T cells pulsed with PHA represent a positive proliferation control

After 7 days of co-cultivation with imDCs or mDCs pulsed with the different OVA formulations, the absolute number of CD4^+^ T cells was assessed(Fig. 7a and b). As expected, the number of CD4^+^ T cells was significantly higher when co-cultured with mDCs and no significant difference was found among the different antigen formulations. The only major difference was observed in the co-culture with mDCs pulsed with 5X dose of OVA-PLGA/PEI NPs, in which the absolute number of CD4^+^ T cells was unexpectedly almost 2 logs lower than the others (5.6x10^3^) (Fig. 7b). The co-cultivation with imDCs showed that the OVA antigen was able to induce a significant increase in the number of CD4^+^ T cells only when formulated in NPs. No significant difference was observed between the two NP formulations. (Fig. 7a).

In addition, the percentage of proliferating cells was evaluated as further parameter (Table 2). Immature DCs induced proliferation of 31.9 and 24.8 % of CD4^+^ T cells when loaded with OVA-PLGA NPs at 1X and 5X dose, respectively. This was approx. a 2 fold increase compared to the proliferation induced by imDCs pulsed with the same doses of soluble OVA (17.4 and 12.8 %, respectively). Such an increase in proliferation was not observed in CD4^+^ T cells co-cultivated with imDCs loaded with either doses of OVA-PLGA/PEI NPs (17.1 and 5.0 %, respectively). Indeed, the 5X dose of OVA-PLGA/PEI NPs seemed to have an inhibitory effect on the overall CD4^+^ T cell proliferation.

**Table 2:** Percentage of proliferating CD4^+^ T cells co-cultivated with imDCs or mDCs

Treatment	Antigen presenting cells	
	imDCs	mDCs
Null	1.7 %	0.1 %
OVA	17.4 %	32.0 %
OVA 5X	12.8 %	27.0 %
OVA 500X	22.4 %	28.9 %
OVA-PLGA	31.9 %	35.3 %
OVA-PLGA 5X	24.8 %	34.4 %
OVA-PLGA/PEI	17.1 %	16.0 %
OVA-PLGA/PEI 5X	5.0 %	15.9 %
PHA	26.2 %	27.4 %

On the contrary, mDCs induced similar levels of proliferating CD4^+^ T cells when loaded with either OVA-PLGA NPs or soluble OVA at both 1X and 5X doses, without any significant increment given by the antigen encapsulation in NPs (above 30 %). Also for mDCs, PLGA/PEI OVA NPs seemed to have an inhibitory effect on the overall CD4^+^ T cell proliferation at both doses (approx. 16.0 %).

Naïve unstimulated CD4^+^ T cells showed 0.1 % basal activation, whereas CD4^+^ T cells pulsed with PHA showed a 27.4 % proliferation.

### Effect of NPs on *ex vivo* differentiation of naïve CD4^+^ T cells

Proliferating CD4^+^ T cells induced by co-cultivation with imDCs as well as mDCs were fully characterized for their differentiation pattern. As expected, OVA antigen presentation by imDCs to CD4^+^ T cells induced a progressive reduction of CD45RA^+^/CD45RO^−^/CD62L^+^ naïve T cells, with a complementary increase of the effector memory and central memory T cells (Fig. 7c). Such an effect was more evident when OVA antigen was delivered to imDCs in both NP formulations. Similarly, OVA antigen presentation by mDCs to CD4^+^ T cells induced a progressive reduction of CD45RA^+^/CD45RO^−^/CD62L^+^ naïve T cells with a complementary increase of the effector memory and central memory T cells (Fig. 7d). However, such an effect was very comparable regardless the way of OVA antigen delivery to DCs (i.e., soluble or in NP formulation). Overall, OVA presentation by imDCs and mDCs was equally effective in inducing effector T cells. In particular, effector memory T cells were more efficiently induced by imDCs, whereas central memory T cells were more efficiently induced by mDCs.

### Effect of NPs on cytokine secretion

**Fig. 8:**
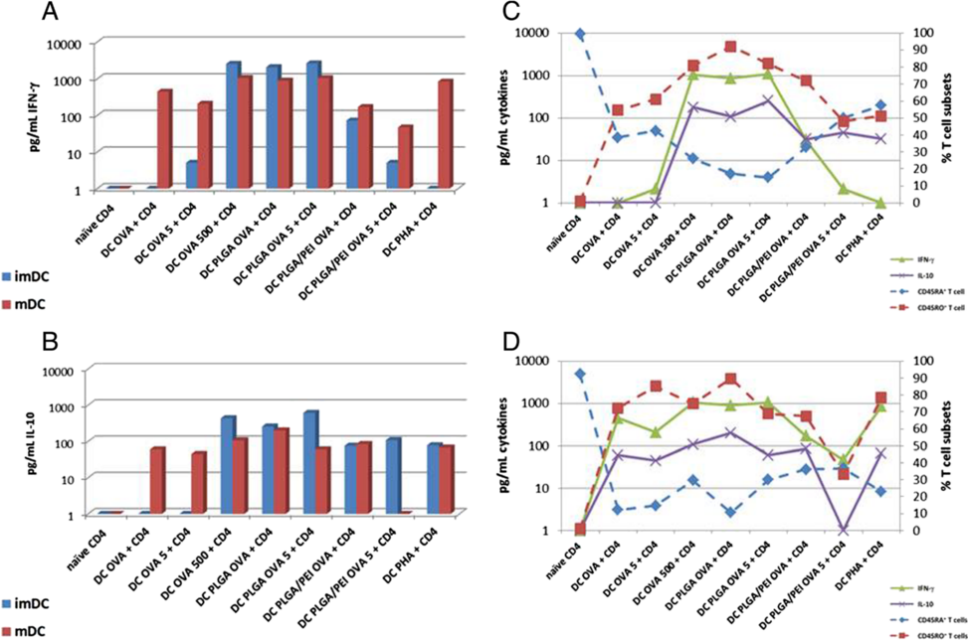
Effect of NPs on cytokine secretion. IFN-γ (**a**) and IL-10 (**b**) production was evaluated after 7 days of T cell co-cultivation with either OVA-imDCs or OVA-mDCs. A correlation analysis between CD4^+^ T cell subsets and cytokine production was performed in either OVA-imDCs (**c**) or OVA-mDCs (**d**) co-culture setting

IFN-γ and IL-10 production was evaluated in cell supernatants at the end of the 7 days co-culture. In particular, CD4^+^ T cells co-cultured with imDCs loaded with soluble OVA did not produce any detectable cytokine level unless OVA was delivered at a 500X dose (Fig. 8a and b). On the contrary, imDCs loaded with NPs loaded with OVA were extremely effective in inducing both IFN-γ and IL-10 production by CD4^+^ T cells (IFN-γ > IL-10). In particular, either doses of OVA-PLGA NPs induced 2–3 log increase in cytokine production over the same doses of OVA as soluble antigen. The 1X dose of OVA-PLGA/PEI NPs was less effective but still induced an approx. 100 fold increase in cytokine production compared to the same dose of soluble OVA. Interestingly, the 5X dose of OVA-PLGA/PEI NPs did not increase IFN-γ production (Fig. 8a).

On the other hand, CD4^+^ T cells stimulated by mDCs produced very high levels of both cytokines (IFN-γ > IL-10) with a limited 2–4 fold increase provided by NPs vs. soluble delivery of OVA antigen. Indeed, the highest production was observed when mDCs were stimulated by either the 500X dose of soluble OVA or the 1X and 5X doses of OVA-PLGA NPs. Interestingly, either doses of PLGA/PEI OVA NPs did not induce an increase in cytokine production compared to corresponding dose of soluble antigen (Fig. 8a and b).

In addition, to determine which subset of CD4^+^ T cells (i.e., naïve T cells, effector T cells, central memory, effector memory) was the major contributor to the observed cytokine production, a correlation analysis was performed between the percentage of distinct T cell phenotypes and the cytokine levels.

The correlation analysis was performed combining the four subsets in two distinct groups: the CD45RA^+^ (naïve and effector T cells) and the CD45RO^+^ (effector memory and central memory T cells) cells (Fig. 8c and d). In this setting, the analysis clearly showed a very strong direct correlation between the pattern of CD45RO^+^ T cells and cytokine production with almost superimposable curves in the mDCs setting (correlation coeff. 0.62; *p* = 0.01) (Fig. 8d). Similarly, the pattern of CD45RA^+^ cells and cytokine production showed a very strong inverse correlation (correlation coeff. –0.47 *p* = 0.01) (Fig. 8d).

## Discussion

A functional characterization of biodegradable NPs based on PLGA modified with PEI (PLGA/PEI) was performed in the present study. Their property as carriers to promote Ag presentation in human DCs was compared to plain PLGA NPs. Unloaded and OVA-loaded PLGA and PLGA/PEI NPs showed very similar size with opposite zeta potential (Table 1). Both NPs entrapped OVA with high encapsulation efficiency. However, PLGA/PEI NPs exhibited a tendency to aggregation in the presence of FBS-enriched cell culture media as previously found for PEI-coated PLGA NPs [18].

NP uptake was evaluated in mouse Hepa 1–6 continuous cell line and in primary human PBMCs at different concentrations and time points (Fig. 3). The uptake profile of PLGA-Rhod/PEI NPs, analyzed by flow cytometry, showed saturation soon after 15 min of incubation with very high levels of fluorescent cells (Fig. 3c and d). On the contrary, the uptake of PLGA-Rhod NPs by both cell types showed a dose- and time-dependent increasing profile, reaching the saturation level only after 24 h of incubation (Fig. 3a and b). Such a result could be explained by the positive surface charge of PLGA-Rhod/PEI NPs which facilitated adherence to negatively charged cellular membranes, increasing intracellular uptake [19]. These results were further confirmed by fluorescence microscopy as well as CLSM analysis (Figs. 4 and 5). Indeed, PLGA-Rhod/PEI NPs showed a marked trend to aggregation with a massive cellular internalization associated with a clear pattern of cellular clustering. PLGA-Rhod NPs showed progressive internalization in both cell types. A lack of correlation between the two measurements was not surprising given that the number of Rhodamine-labeled NPs entering each cell greatly varied.

TEM analysis confirmed a significantly different pattern of intracellular localization according to the type of NPs loaded on the cells. Indeed, while PLGA/PEI NPs were taken up as multiple particles and reached the perinuclear area (Fig. 6d - f), PLGA NPs were taken up as single particles and remained localized at the cellular apical area (Fig. 6a - c). Both NPs showed an internalization pathway resembling clathrin-dependent (Fig. 6g and i) as well as a caveolin-dependent (Fig. 6j and k) pathways [15]. Moreover, PBMCs loaded with PLGA/PEI NPs often showed interactions between vesicles and nuclear membrane (Fig. 6l). The latter observation is extremely interesting because it has never been reported in literature [20].

Finally, the efficacy of antigen delivery by PLGA and PLGA/PEI NPs to antigen-presenting cells was assessed in an *ex vivo* priming assay. Results showed that imDCs loaded with 1X dose of OVA formulated in both NP formulations induced the duplication of CD4^+^ T cells more efficiently than soluble OVA (even at 500X dose) (Fig. 7a). On the contrary, T cell duplication was induced by mDCs at same levels when OVA was delivered to DCs as soluble antigen or encapsulated in both types of NPs (Fig. 7b).

Such a result strongly indicates that the 1X dose of OVA formulated in NPs is able to induce a maturation of imDCs and subsequent activation of co-cultured autologous naïve CD4^+^ T cells. On the contrary, OVA-PLGA/PEI NPs, and in particular the 5X dose, seemed to have an inhibitory effect on the overall CD4^+^ T cell proliferation. This unexpected result is currently under investigation.

Overall, the effect elicited was stronger when OVA was encapsulated in mDCs than in imDCs, showing that PLGA NPs are not able to induce a full maturation pattern of DCs even in the presence of PEI. This strongly supports the need for additional functionalization in order to provide NPs with the full adjuvanting effect.

Differentiation of naïve CD4^+^ T cells varied according to antigen delivery to imDCs and mDCs. Indeed, the most evident increase in effector memory and central memory T cells was observed when OVA antigen was delivered to imDCs in NP formulations (Fig. 7c). On the contrary, no striking difference in CD4^+^ T cell differentiation was observed when OVA antigen was delivered to mDCs either soluble or encapsulated in NPs (Fig. 7d). In both experimental settings, the highest percentage of central memory T cells was induced by the 1X dose of OVA-PLGA NPs. The highest percentage of effector memory T cells was induced by the 5X dose of OVA-PLGA NPs, in the imDC setting, and by the 500X dose of soluble OVA in the mDC setting. Conversely, in both experimental settings, the highest percentage of effector T cells was induced by the 5X dose of OVA-PLGA/PEI NPs. Such results provide evidence that either imDCs or mDCs when loaded with 1X dose of OVA formulated in PLGA NPs are extremely effective in skewing naïve CD4^+^ T cells toward the full spectrum of activated T cell phenotypes, including central memory T cells which are crucial for the establishment of immunological memory.

The apparent discordance between efficient uptake and antigen presentation to T cells observed for PLGA/PEI NPs, could be explained by the strong interaction occurring between PEI and OVA. This could significantly reduce the OVA protein release and its availability for processing and presentation in the context of MHC II.

The evaluation of cytokines produced in the co-cultures indicated a strong induction of a Th1 phenotype, characterized by high levels of IFN-γ production and lower levels of IL-10 (Fig. 8a and b). Such CD4^+^ T helper phenotype is suggestive of an effective CD8^+^ T cell response, as previously shown for PLGA NPs [8, 21]. Only OVA encapsulated in NPs was able to induce same levels of cytokines when presented to both imDCs and mDCs. The modification with PEI did not enhance the effect of PLGA NPs but even reduced it. Additional studies are in progress to dissect such an effect. Soluble OVA induced the same effect in both DCs only at the 500 X dose, at lower doses the effect was observed in mDCs only.

The present results are in agreement with the evidence that Th1 cells may produce both IFN-γ and its negative regulator IL-10 as negative feed-back loop to contain an excessive immune response [22, 23].

Furthermore, a correlation analysis showed that, indeed, the CD45RO^+^ memory T cells were the major if not the only contributor to production of both cytokines (Fig. 8 c and d), in agreement with previous studies [24], suggesting the efficient induction by NPs of memory CD4^+^ Th1 cells.

## Conclusions

Biodegradable nanoparticles represent a very effective antigen delivery system, providing sufficient co-stimulatory signals able to induce innate as well as effector/memory adaptive immune responses with limited antigen dose. The side-by-side comparison showed that PLGA/PEI NPs, although providing higher cell uptake in imDCs, are comparable to PLGA NPs in delivering the OVA non-self antigen to DCs, inducing differentiation of autologous naïve CD4^+^ T cells into memory cells with a Th1 phenotype. In our experimental setting, modification with PEI may represent a promising approach in therapeutic cancer vaccine strategy development, but it does not appear to enhance the antigen presentation activity of PLGA NPs. Indeed, further optimization of the model is required to parallel effects obtained with NPs decorated with ligands of DC receptors.

## Methods

### Materials

Poly (D,L-lactide-co-glycolide) (PLGA) (50:50 Resomer RG 502H inherent viscosity 0.16–0.24 dL/g) was purchased from Boehringer Ingelheim (Ingelheim, Germany). Polyethyleneimine (PEI, MW = 10–25 kDa branched), poloxamer 188 (Pluronic® F68), Bradford reagent, potassium phosphate dibasic and potassium phosphate monobasic, sodium azide, sodium chloride, copper (II) sulphate and albumin from chicken egg white (OVA), were purchased from Sigma-Aldrich. Sodium hydroxide was provided from Delchimica Scientific Glassware. Ethanol (96 %), phosphoric acid (85 %), acetic acid, acetonitrile and tetrahydrofuran were purchased from Carlo Erba Reagenti (Milan, Italy).

### Preparation of NPs

Unloaded and OVA-loaded PLGA and PLGA/PEI NPs were prepared by a modified emulsion-solvent diffusion technique [25]. An aqueous solution containing 1 mg of OVA (50 μL) was emulsified by an ultrasound probe for 2 min at 3W (Sonicator 3000, Misonix, USA) with 1.5 mL of PLGA (1.5 % w/v) and poloxamer 188 (1.5 % w/v) dissolved in methylene chloride. The resulting emulsion was quickly added to 7.5 mL of ethanol, leading to an immediate precipitation of the polymer in the form of NPs. The dispersion was diluted with 7.5 mL of ultrapure water and stirred at RT for 1 h. The organic solvents were evaporated under vacuum at 30 °C. NPs were washed twice with ultrapure water under centrifugation at 13220 x g for 15 min. The pellet was re-dispersed in water and freeze-dried for 24 h (addition of threalose 4:1 mass ratio to polymer weight). Recovery yield of production process was evaluated on an aliquot of NP dispersion by weighting the solid residue after freeze-drying. Results are expressed as the ratio of the actual NPs weight to the theoretical polymer weight x 100. Fluorescent NPs were prepared analogously by incorporating a 20 % w/w of PLGA-Rhod to PLGA NP core [26].

### Characterization of NPs

The hydrodynamic diameter (D_H_) and polydispersity index (PI) of NPs were determined by Photon Correlation Spectroscopy (PCS) using a N5 Submicron Particle Size Analyzer (Beckman-Coulter). A NP dispersion was diluted in Milli-Q water at intensity in the range 10^4^–10^6^ counts/s and measurements were performed at 25 °C on 90° angle. Results are reported as mean D_H_ of three separate measurements on three different batches ± SD. Size of NPs stored in the dark at 4 °C for 3 months was monitored too.

Zeta potential was determined by analyzing a NP dispersion in water on a Zetasizer Nano Z (Malvern Instruments Ltd.). Results are reported as mean of three separate measurements of three different batches (*n* = 9) ± SD.

### PEI amount associated to NPs

PEI was quantified by a colorimetric method developed by us. To this purpose, 0.5 mg of freeze-dried PLGA/PEI NPs were treated with 0.5 mL of 1 M NaOH and stirred overnight. The sample (0.5 mL) was diluted with 0.5 mL of 1 M acetic acid. The resulting solution (0.5 mL) was added to 1 mL of acetate buffer 0.1 M at pH 4.5 and complexed with 0.25 mL of a copper (II) sulphate water solution 0.1 % w/v. The absorbance value of each solution was recorded at 281 nm (UV 1800, Shimadzu). A calibration curve was constructed in the same condition in the PEI concentration range 15–380 μg/mL. Possible interference of PLGA on PEI quantitative analysis was assessed by treating and analyzing a similar amount of unloaded PLGA NPs in the same conditions.

### OVA entrapment efficiency

OVA loading inside NPs was assessed by dissolving 1 mg of NPs in 500 μL of methylene chloride under stirring for 15 min. Thereafter, 500 μL of water were added and after stirring for further 15 min, the sample was centrifuged at 2000 x g for 5 min. The supernatant (0.1 mL) was collected and added to 0.9 mL of Bradford reagent. After incubation for 15 min, the absorbance of the solutions was measured at 595 nm on an UV spectrophotometer (UV 1800, Shimadzu) and compared to a calibration curve generated in the OVA concentration range 20–1000 μg/mL. To verify a possible interference of copolymers on OVA quantitative analysis, an amount of unloaded NPs was treated and analyzed in the same conditions.

### NP stability in cell culture media

The stability of NPs under physiologically relevant conditions was evaluated by placing a known amount of NPs in DMEM (Dulbecco’s Modified Eagle Medium, Life Technologies) and RPMI 1640 (Life Technologies) cell culture media supplemented with FBS 10 % (NP concentration was 1.5 mg/mL) at 37 °C. Size measurements of the samples were taken by PCS after 24, 48 and 72 h of incubation.

### Nanoparticle uptake

Hepa 1–6 or PBMCs were seeded into each well of a 24-well culture plate in a maximum volume of 1 mL/well for induction. PLGA NPs labeled with rhodamine B were added to the culture medium 24 h later. The final NPs concentration was adjusted to 20, 50 and 100 μg/mL. Following different incubation times, the cells were washed with 1X PBS and further treated for FACS and CLSM analysis.

### FACS analysis

Hepa 1–6 cells were washed twice with 1X PBS and studied with a FACScalibur flow cytometer (BD FACSCanto II). In parallel, NP-loaded PBMCs were stained with PerCP-Cy5.5-anti-human CD14 antibody for 30 min at 4 °C before analysis.

### CLSM analysis

Confocal laser scanning microscopy (CLSM) was applied to image the Hepa 1–6 and PBMCs after incubation with NPs. Cells (2x10^4^/sample) were seeded on sterile glass coverslips and cultured in suitable growth medium. 24 h later, fluorescently labeled NPs were added to the culture medium with a final concentration of 50 μg/mL. Following different incubation times, the culture medium was removed and the cells were rinsed with 1X PBS. Cover slips were mounted using 20 % (w/v) mowiol (Sigma-Aldrich). Cells were visualized with a 510 META-LSM confocal microscopy (Carl Zeiss) and z-series with 0.1 μm intervals were collected. Alternatively, after nuclear staining with 4-6-diamidino-2-phenylindole dye (DAPI), cells were visualized by a fluorescence inverted microscope connected to a video-camera (Carl Zeiss).

### TEM analysis

50 μl of each NP dispersion was applied to a carbon-coated copper/rhodium grid (400 mesh) (TAAB Laboratories Equipment Ltd, Aldermaston, Berks, ENGLAND). The coated grid was floated for 2 min on the drop, rinsed with 200 μl of double distilled water and negative stained by 200 μl of 2 % w/v uranyl acetate solution (TAAB Laboratories Equipment Ltd). After draining off the excess of staining solution, the specimen was transferred to the electron microscope for examination, using a TEM-Philips Morgagni 282D transmission electron microscope, operating at 60 kV. Cells incubated with NPs at 15 min, 30 min and 24 h were processed according to embedding standard procedures, i.e., fixation in 2.5 % glutaraldehyde in 1X PBS for 2 h, post-fixation at 4 °C in 1 % osmium tetroxide in the same buffer for 1 h, bulk staining overnight in 0,5 % aqueous uranyl acetate, dehydration in graded ethanol dilutions, and embedding in TAAB Spurr resin (TAAB Laboratories Equipment Ltd, Aldermaston, Berks, ENGLAND). Diamond thin sections were stained with lead citrate before TEM observations at 80 KV accelerating voltage. All the electron micrographs were photographed on Kodak electron microscope film 4489 (Kodak Company, New York, USA).

### Human PBMC isolation and DC generation

Fresh human PBMCs were isolated, from EDTA-treated blood collected from healthy donors, by Ficoll-Hypaque density gradient centrifugation and plated in 6-well plates at a concentration of approximately 1 × 10^6^cells/well in a maximum volume of 2 mL/well.

Monocyte-derived dendritic cells (MDDCs) were generated as described previously, with minor modifications [17]. Briefly, isolated PBMCs were allowed to adhere to plastic in 6-well plates for 2 h. Adherent monocytes were washed with RPMI 1640 medium and were then cultured for 6 days in DC culture medium supplemented with 50 ng of recombinant granulocyte-macrophage colony-stimulating factor (rGM-CSF; R&D Systems, Minneapolis, Minn.) per mL and 1000 U of recombinant interleukin-4 (rIL-4; R&D Systems, Minneapolis, Minn.) per mL. Immature DCs (imDCs) were ready for NP treatment.

### Isolation of naïve CD4^+^ T cells

Naïve CD4^+^ T cells were isolated from PBMCs by negative selection with a mixture of mAbs from StemCell Technologies (Vancouver, British Columbia, Canada), according to the manufacturer’s instructions. Anti-human HLA-DR tetrameric mAb (StemCell Technologies) was also included to increase purity of naïve CD4^+^ T cells.

### Antigen-specific stimulation of autologous naïve CD4^+^ T cells *ex vivo*

Human imDC were pulsed O/N with 1.55 and 7.75 μg OVA, either soluble or encapsulated in NPs. Cell viability was assessed by MTT assay according to the manufacturer’s instructions (Sigma). OVA-treated imDCs (OVA-imDCs) were used directly or stimulated for additional 48 h with LPS (200 ng/mL) to induce DC maturation (OVA-mDCs). OVA-imDCs and OVA-mDCs were co-cultured for 7 days with autologous naïve CD4^+^ T cells labeled with carboxyfluorescein succinimidyl ester (CellTrace™ CFSE Cell Proliferation Kit; Life Technologies), according to the manufacturer’s instructions. Briefly, 5x10^4^ DCs/per well were used for priming autologous naïve CD4^+^ T cells (2x10^5^ cells/per well) in 200 μl of total culture volume in 96-well plates. The co-cultures were incubated in a humidified incubator in the presence of 5 % CO_2_ at 37 °C for 7 days. T cell proliferation and differentiation was estimated by flow cytometry by cell count using antibodies directed against CD4, CD62L, CD45RO and CD45RA, and CFSE dilution. After 7 days, supernatants from the assay wells were removed and IFN-γ as well as IL-10 were measured with the Platinum ELISA kit (eBioscience), according to the manufacturer’s instructions.

### Statistical analyses

Data were analyzed by descriptive statistics, calculating the mean and standard deviation for continuous variables.

## Additional file

Additional file 1: Table S1. Composition and properties of fluorescent unloaded NPs. SD were calculated on three different batches. (DOCX 17 kb)

## Abbreviations

APCs: Antigen-presenting cells
DCs: Dendritic cells
NPs: Nanoparticles
PLGA: Poly (D,L-lactide-co-glycolide)
PEI: Polyethylenimine
CLSM: Confocal laser scanning microscopy
TEM: Transmission electron microscopy
MVE: Multivesicular endosomes
OVA: Ovalbumin
imDCs: Immature dendritic cells
mDCs: Mature dendritic cells
Th1: T helper 1
CTL: Cytotoxic T lymphocyte
PBMCs: Peripheral blood mononuclear cells
MFI: Mean fluorescence intensity
MDDCs: Monocyte-derived dendritic cells
GM-CSF: Granulocyte-macrophage colony-stimulating factor
IL-4: Interleukin 4
IL-10: Interleukin 10
IFN-γ: Interferon-gamma
LPS: Lipopolysaccharide
D_H_: Hydrodynamic diameter
PI: Polydispersity index
PCS: Photon correlation spectroscopy
FACS: Fluorescence activated cell sorting
