# Supplementary material for: Functional characterization of biodegradable nanoparticles as antigen delivery system
Source: J Exp Clin Cancer Res. 2015 Oct 6;34:114. doi: 10.1186/s13046-015-0231-9 (PMC4596393; doi:10.1186/s13046-015-0231-9)
Supplement: Additional file 1: Table S1. — Composition and properties of fluorescent unloaded NPs. SD were calculated on three different batches. (DOCX 17 kb) [file 13046_2015_231_MOESM1_ESM.docx]

**Additional file 1**

**Table S1.** Composition and properties of fluorescent unloaded NPs. SD were calculated on three different batches.

| **Code** | **Mean D_H_**  **(nm±SD)** | **P.I.** | **Zeta Potential**  **(mV±SD)** | **PEI**  **Actual loading**  **(mg/100 mg NPs)** |
| --- | --- | --- | --- | --- |
| PLGA-Rhod NPs | 199 ± 7 | 0.093 | -34 ± 6 | - |
| PLGA-Rhod/PEI NPs | 196 ± 9 | 0.135 | +36 ± 3 | 6.6 |
